# Prevalence of Germline Pathogenic and Likely Pathogenic Variants in Patients With Second Breast Cancers

**DOI:** 10.1093/jncics/pkaa094

**Published:** 2020-10-26

**Authors:** Katharine A Yao K, Jacob Clifford, Shuwei Li, Holly LaDuca, Peter Hulick, Stephanie Gutierrez, Mary Helen Black

**Affiliations:** 1 Department of Surgery, NorthShore University HealthSystem, Evanston, IL, USA; 2 Ambry Genetics, Aliso Viejo, CA, USA; 3 Department of Medicine, Center for Medical Genetics, NorthShore University HealthSystem, Evanston, IL, USA

## Abstract

**Background:**

Few studies have examined gene-specific associations with contralateral and/or second breast cancer (SBC).

**Methods:**

The frequency of pathogenic and likely pathogenic (P/LP) variants in clinically actionable genes (*BRCA1*, *BRCA2*, *PTEN*, *TP53*, *CHEK2*, *CDH1*, *ATM*, *PALB2*, *NBN*, and *NF1*) was compared between women with a primary breast cancer (PBC) and SBC who underwent multigene panel testing at a single diagnostic testing laboratory. Race- and ethnicity-specific logistic regression burden tests adjusted for age at diagnosis of first breast cancer, histology, presence of first- or second-degree relatives with breast cancer, and prior testing for *BRCA1/2* genes were used to test for associations with SBC. All statistical tests were 2-sided.

**Results:**

The study was comprised of 75 550 women with PBC and 7728 with SBC. Median time between breast cancers for SBC was 11 (interquartile range = 6–17) years. Restricting to women tested for all actionable genes (n = 60 310), there were 4231 (7.8%) carriers of P/LP variants in actionable genes among the controls (PBC) compared with 652 (11.1%) women with SBC (*P*< .001). Among Caucasians, exclusive of Ashkenazi Jewish women, those carrying a P/LP variant in a clinically actionable gene were 1.44 (95% confidence interval [CI] = 1.30 to 1.60) times as likely to have SBC than noncarriers, after accounting for potential confounders. Among African American and Hispanic women, a P/LP variant in a clinically actionable gene was 1.88 (95% CI = 1.36 to 2.56) and 1.66 (9% CI = 1.02 to 2.58) times as likely to be associated with SBC, respectively (*P* < .001 and *P* = .03).

**Conclusion:**

Women with P/LP variants in breast cancer predisposition genes are more likely to have SBC than noncarriers. Prospective studies are needed confirm these findings.

Women who have undergone bilateral mastectomy for breast cancer often state that worry about developing a contralateral breast cancer (CBC) or a second breast cancer (SBC) was one of the main reasons they underwent bilateral mastectomy (1-3). Genetic testing can help clarify risk for SBCs and is frequently recommended for newly diagnosed breast cancer patients to inform surgical decisions. Studies have shown that newly diagnosed breast cancer patients who have undergone genetic testing are more likely to undergo bilateral mastectomy (4-6) especially if a pathogenic and likely pathogenic variant is identified. However, in recent years, the clinical genetic testing approach for hereditary breast cancer has shifted from single to multigene panel testing, resulting in the increased identification of patients with pathogenic variants in predisposition genes beyond *BRCA1/2* (7-9). Guidelines recommend consideration of bilateral mastectomy for women newly diagnosed with breast cancer carrying pathogenic variants in *BRCA1* or *BRCA2* based on 20-year cumulative CBC risks of 40% and 26%, respectively (10-14), but recommendations for women with pathogenic or likely pathogenic (P/LP) variants in breast cancer predisposition genes other than *BRCA1* or *BRCA2* are not available, because cumulative long-term risks of a SBC are not well established*.* Although some studies have suggested a relationship between P/LP variants in genes such as *CHEK2* and *PALB2* and higher SBC risk (15,16), they were not sufficiently powered to reliably confirm an association.

To examine the association between P/LP variant in clinically actionable variants (according to the National Comprehensive Cancer Network [NCCN] Clinical Practice Guidelines in Oncology [NCCN Guidelines]) with SBC or CBC, we conducted a retrospective analysis of nearly 90 000 women referred for genetic testing at a single diagnostic laboratory. The objective of this study was to provide robust estimates of the prevalence of P/LP variants among patients with a CBC or SBC in lieu of a long-term longitudinal study. We compared the odds of a SBC in women who carry clinically actionable NCCN P/LP variants compared with women who do not carry any P/LP variants. Although treatment information was not available, we controlled for other potential confounders of SBC such as age at PBC diagnosis, family history, and prior *BRCA1/2* testing to determine the association of P/LP variants in all “clinically actionable” genes with SBC. Our large dataset also enabled us to examine whether the prevalence of these variants differed by race and ethnicity after accounting for patient and tumor characteristics.

## Methods

### Patient Population

The study population consisted of female breast cancer patients who underwent multigene panel testing at Ambry Genetics from March 2012 to December 2016 (n = 87 229). Demographic, family history, and clinical information (sex, age, self-reported ethnicity, personal cancer history, age at diagnosis, and breast tumor characteristics) were collected from test requisition forms provided by ordering clinicians, as well as other clinical documentation if provided. Women with potential synchronous breast cancer were identified as those who had a SBC diagnosis within 1 year of the first diagnosis (n = 3951) and were excluded from primary analyses. This study did not use patient identifier information, and institutional review board approval was deemed exempt by NorthShore University Health System.

### Multigene Panel Testing

Patients underwent comprehensive sequencing of *BRCA1/2* and other cancer predisposition genes (Supplementary Table 1, available online), as previously described (17,18). Variants were assessed using Ambry’s 5-tier classification framework based on guidelines published by the American College of Medical Genetics and Genomics and the Association for Molecular Pathology (pathogenic, likely pathogenic, variant of uncertain significance, likely benign, benign) (19,20). The classification framework incorporates multiple lines of evidence such as functional and structural impact, evolutionary conservation, allele frequency in the general population, co-segregation, case-control data, and phenotype (17).

### Pathogenic and Likely Pathogenic Variants

P/LP variants were both considered positive test results. P/LP variants in clinically actionable genes, defined as those with recommendations for increased breast cancer screening and/or risk reduction by the NCCN Guidelines for Genetic/Familial High-Risk Assessment: Breast, Ovarian, and Pancreatic (21) were examined (*BRCA1*, *BRCA2*, *TP53*, *PTEN*, *ATM*, *CHEK2*, *PALB2*, *CDH1*, *NBN*, *NF1*). Clinically actionable genes were further grouped based on recommendations surrounding risk-reducing mastectomy (RRM): “discuss option of RRM” (RRM^+^) includes *BRCA1*, *BRCA2*, *TP53*, *PALB2*, and *PTEN*, and those which NCCN qualifies as “evidence insufficient, manage based on family history” (RRM^-^) include *ATM*, *CHEK2*, *CDH1*, *NBN*, and *NF1*.

### Statistical Analysis

Differences between PBC and SBC patient and tumor characteristics were assessed with Fisher exact test for categorical variables and 2-sample *t* tests or analysis of variance for continuous variables, as appropriate. The proportion of patients who tested positive for a P/LP in a clinically actionable gene was assessed for the PBC and SBC groups. Patients were included in an analysis of a gene set (eg, RRM^+^) only if they were tested for all genes in the subset. Logistic regression tests stratified by race and ethnicity were used to test for SBC associations with P/LP in actionable gene subsets, adjusting for age at PBC diagnosis, PBC histology, presence of first- or second-degree relative with breast cancer, prior *BRCA1*/*2* testing, and personal history of other nonbreast cancers. Logistic regression was also used to test for SBC association with *CHEK2* c.1100delC specifically, adjusting for all covariates. To additionally control for confounding in the design, women with SBC were matched to those with PBC based on all covariates described above, with PBC diagnosis age categorized in 5-year intervals, and conditional logistic regression was used to estimate associations with SBC. Lastly, we also performed stratified analysis by age at diagnosis of first breast cancer (younger than 50 years vs 50 years and older) among Caucasian patients adjusting for all covariates (except residual age at PBC diagnosis). Race- and ethnicity-specific associations were reported only if carrier counts were 5 or more in both PBC and SBC groups. Sensitivity analyses were conducted to evaluate whether the inclusion of SBC cases with potentially synchronous cancers or restriction of SBC cases to those confirmed to have CBC or exclusion of ductal carcinoma in situ cases influenced the observed estimates of association. We further performed additional analyses matching women with PBC and SBC on time between age at first breast cancer diagnosis and age at genetic testing (5-year interval), as well as comparisons of SBC to PBC in which cases with more than 2 breast cancers were excluded from the SBC group (n = 385). Missing values were included as a distinct category so that all observations could be included in the aforementioned analyses.

All statistical analyses were conducted with R v.3.2 (22). *P* values less than .05 were considered statistically significant. Tests of statistical significance were 2-sided.

## Results

### Patient Demographics for PBC and SBC

Among women with PBC (n = 75 550) or SBC (n = 7728), after exclusion of potential synchronous cases, those with SBC were slightly more likely to be Caucasian (*P* <.001; Table^1^). SBC cases tended to be older than PBC when referred for genetic testing (mean age at testing = 63.1 [SD = 10.3] vs 53.4 [SD = 12.1] years; *P* <.001) and slightly younger at first breast cancer diagnosis than PBC (mean age at diagnosis: 47.4 [SD = 10.1] vs 49.5 [SD = 11.5] years; *P* <.001). When stratified by race and ethnicity, Caucasian and Ashkenazi women tended to be older at the time of genetic testing and slightly older at first breast cancer diagnosis and were more likely to have personal history of other cancer primaries and/or first-degree relatives with breast cancer, compared with most other racial and ethnic groups (Supplementary Table 2, available online).

**Table 1.** Patient demographic factors between patients with primary breast cancer and patients with second primary breast cancer

Demographic factor	PBC (n = 75 550)	SBC (n = 7728)	*P*
Race/Ethnicity, No. (%)			<.001
Caucasian	47 884 (63.4)	5238 (67.8)	
Ashkenazi Jewish	3996 (5.3)	468 (6.1)	
African American	5727 (7.6)	642 (8.3)	
Hispanic	4592 (6.1)	254 (3.3)	
Asian	3682 (4.9)	288 (3.7)	
Other/Unknown	9669 (12.8)	838 (10.8)	
Age at testing, mean (SD), y	53.4 (12.1)	63.1 (10.3)	<.001
Age at diagnosis of first breast cancer primary, mean (SD), y	49.5 (11.5)	47.4 (10.1)	<.001
Time between breast primaries, mean (SD), y	—	12.4 (7.7)	—
Panel test, No. (%)			<.001
BRCAplus/BRCAplus expanded	17 464 (23.1)	1462 (18.9)	
GYNplus	1864 (2.5)	163 (2.1)	
BreastNext	23 962 (31.7)	2661 (34.4)	
OvaNext	12 547 (16.6)	1339 (17.3)	
PancNext	216 (0.3)	15 (0.2)	
CancerNext/CancerNext expanded	19 496 (25.8)	2088 (27.0)	
Other	1 (0.0)	0 (0.0)	
Previously tested for BRCA1/2, No. (%)			<.001
Yes	12 281 (16.3)	1967 (25.5)	
No	59 693 (79.0)	5503 (71.2)	
Unknown	3576 (4.7)	258 (3.3)	
Patient-reported second primary type, No. (%)			—
Contralateral/bilateral	—	3880 (50.2)	
Ipsilateral	—	783 (10.1)	
Not provided	—	3063 (39.6)	
Personal history of other cancer, No. (%)^a^			<.001
Yes	8353 (11.1)	1137 (14.7)	
No	67 197 (88.9)	6591 (85.3)	
Personal history of cancers by type, No. (%)			
Ovarian	1371 (1.8)	133 (1.7)	.59
Endometrial	1398 (1.9)	230 (3.0)	<.001
Colorectal	1025 (1.4)	141 (1.8)	.001
Melanoma	1308 (1.7)	176 (2.3)	<.001
Pancreatic	278 (0.4)	31 (0.4)	.62
Other	3349 (4.4)	490 (6.3)	<.001
Timing of personal history for breast with respect to endometrial cancer,^b^ No. (%)			
BC diagnosed before endometrial	638 (45.6)	173 (75.2)	<.001
Endometrial cancer diagnosed before BC	612 (43.8)	45 (19.6)	<.001
BC and endometrial cancers diagnosed in the same year	148 (10.6)	12 (5.2)	.02
Family history of any cancer, No. (%)			
>1 first-degree relative	49 892 (66.0)	5721 (74.0)	<.001
>1 second- or third-degree relatives only	17 746 (23.5)	1258 (16.3)	
None	7912 (10.5)	749 (9.7)	
Family history of breast cancer, No. (%)			
>1 first-degree relative	26 605 (35.2)	3066 (39.7)	<.001
>1 second- or third-degree relatives only	24 555 (32.5)	2188 (28.3)	
none	24390 (32.3)	2474 (32.0)	
Family history of ovarian cancer, No. (%)			
>1 first-degree relative	3953 (5.2)	367 (4.7)	<.001
>1 second- or third-degree relatives only	7491 (9.9)	626 (8.1)	
none	64 106 (84.9)	6735 (87.2)	
Met testing criteria for BRCA1/2,^c^ No. (%)			.007
Yes	68 271 (90.4)	7056 (91.3)	
No	7279 (9.6)	672 (8.7)	
Met testing criteria for Li-Fraumeni syndrome,^c^ No. (%)			<.001
Yes	7900 (10.5)	1000 (12.9)	
No	67 650 (89.5)	6728 (87.1)	

a Additional breast cancer primaries and nonmelanoma skin cancers not included. BC = breast cancer; PBC = primary breast cancer; SBC = second breast cancer.

b Only reported for patients with a personal history of both breast and endometrial cancer (n = 1628).

c Genetic testing criteria as determined by the National Comprehensive Cancer Network Guidelines for Genetic/Familial High-Risk Assessment: Breast, Ovarian, and Pancreatic V1.2020 (21).

### Tumor Characteristics Between PBC and SBC

The majority of women in both SBC and PBC groups had invasive ductal carcinoma (62.0% and 52.4%, respectively), although a substantial proportion of both groups did not report histology information (23.3% and 31.1%, respectively) (Supplementary Table 3, available online). Only 23.0% of SBC patients reported information on all 3 tumor receptors. Of 3200 (41.0%) SBC patients with information on the estrogen receptor (ER) status of the PBC and SBC, 571 (18.0%) had an ER–negative first and second breast cancer, 351 (11.0%) had an ER-positive first and ER-negative second cancer, 388 (12.0%) had an ER-negative first and ER-positive second breast cancer, and 1890 (59.0%) had an ER-positive first and second cancer. The location of the second breast cancer was reported as bilateral and/or contralateral in 50.2% and ipsilateral in 10.1% of SBC cases, and 39.6% did not specify.

### Prevalence of Pathogenic and Likely Pathogenic Variants in SBC vs PBC

Among all women tested with multigene panels including all clinically actionable breast cancer genes (n = 60 310), 4883 (8.1%) were carriers of at least 1 P/LP variant (11.1% SBC vs 7.8% PBC). Of those tested only for the subset of RRM^+^ (n = 68 822) or RRM^-^ genes (n = 60 311), 3051 (4.4%) and 2421 (4.0%) were carriers of P/LP variants, respectively. The gene with the highest frequency of P/LP variants in both PBC and SBC was *CHEK2* (3.4% SBC vs 2.3% PBC), followed by *BRCA1* (2.7% SBC vs 1.6% PBC), *BRCA2* (2.2% SBC vs 1.8% PBC), and *PALB2* (1.4% SBC vs 0.9% PBC).

### Association Analysis of Pathogenic and Likely Pathogenic Variants in SBC vs PBC by Race and Ethnicity

Among Caucasians, exclusive of Ashkenazi Jewish women, those carrying a P/LP variant in a clinically actionable gene were 1.44 (95% confidence interval [CI] = 1.30 to 1.60) times as likely to have SBC as noncarriers, after accounting for potential confounders (Table 2). Similarly, those with a P/LP variant in RRM+ or RRM- genes were 1.41 (95% CI = 1.22 to 1.62) or 1.36 (95% CI = 1.18 to 1.56) times as likely to have SBC as those without, respectively. *BRCA1*, *CHEK2*, and *BRCA2* were statistically significantly enriched in SBC vs PBC (odds ratio [OR] = 1.56, 95% CI = 1.28 to 1.89; OR = 1.57, 95% CI = 1.30 to 1.88; and OR = 1.33, 95% CI = 1.08 to 1.62, respectively), whereas *NBN* and *PALB2* were associated with SBC at marginal statistical significance (OR = 1.77, 95% CI = 0.97 to 3.02; OR = 1.32, 95% CI = 0.98 to 1.74; *P* = .05 and *P* = .06, respectively). The single variant *CHEK2* c.1100delC also showed an association with SBC (OR = 1.52, 95% CI = 1.18 to 1.93). When SBC cases were matched with PBC patients on potential confounders, similar associations were observed (Supplementary Table 4, available online). Among women with PBC aged younger than 50 years or older than 50 years, those carrying a P/LP variant in an actionable gene were 1.56 (95% CI = 1.37 to 1.78) and 1.32 (95% CI = 1.10 to 1.58) times as likely to develop an SBC as noncarriers (Supplementary Table 5, available online). An analysis of those patients with complete ER status of the PBC showed that women with a NCCN clinically actionable P/LP variant had an odds ratio of 1.41 (95% CI = 1.22 to 1.63) for SBC (data not shown). Heterogeneity tests comparing odds ratios between the early vs late-onset groups were not statistically significant, except for *BRCA1* and *TP53* (*P* = .03 and *P* = .03, respectively). When PBC and SBC women were matched on time between age at first breast cancer and age at testing, adjusted analyses yielded an SBC odds ratio for P/LP variant carriers vs noncarriers of 1.44 (95% CI = 1.26 to 1.64) (Table 3).

**Table 2.** Adjusted odds ratios and 95% confidence intervals for gene associations with SBC among Caucasian patients

Genes or gene group	PBC			SBC			AOR (95% CI)^a^	*P*
	n_carrier_	n_tested_	Mutation prevalence, %	n_carrier_	n_tested_	Mutation prevalence, %		
Group^b^								
All actionable genes	2753	34 648	7.95	465	3979	11.69	1.44 (1.30 to 1.60)	<.001
RRM^+^	1546	39 383	3.93	254	4468	5.68	1.41 (1.22 to 1.62)	<.001
RRM^-^	1518	34 648	4.38	249	3979	6.26	1.36 (1.18 to 1.56)	<.001
Genes								
ATM	489	37 742	1.30	71	4320	1.64	1.15 (0.89 to 1.47)	.28
BRCA1	677	47 884	1.41	124	5238	2.37	1.56 (1.28 to 1.89)	<.001
BRCA2	784	47 884	1.64	112	5238	2.14	1.33 (1.08 to 1.62)	.005
CHEK2^c^	741	37 293	1.99	141	4268	3.30	1.57 (1.30 to 1.88)	<.001
CDH1	—	—	—	—	—	—	—	—
NBN	75	35 723	0.21	15	4136	0.36	1.77 (0.97 to 3.02)	.05
NF1	—	—	—	—	—	—	—	—
PALB2	358	39 561	0.90	55	4481	1.23	1.32 (0.98 to 1.74)	.06
PTEN	—	—	—	—	—	—	—	—
TP53	99	47 852	0.21	20	5234	0.38	1.34 (0.80 to 2.14)	.24

a Odds ratios estimated from models adjusted for age at diagnosis of first breast cancer, histology of the first breast cancer, personal history of other cancer, presence of first- or second-degree relative with breast cancer, and prior *BRCA1* and *BRCA2* genetic testing. “—” indicates gene sets or specific genes for which there were <5 carriers in any group. AOR = adjusted odds ratios; CI = confidence interval; PBC = primary breast cancer; RRM = risk-reducing mastectomy; SBC = second breast cancer.

b RRM^+^: the set of genes recognized by NCCN Guidelines for Genetic/Familial High-Risk Assessment: Breast, Ovarian, and Pancreatic V1.2020 (21) as appropriate for discussion of risk-reducing mastectomy; RRM^-^: the set of genes for which NCCN Guidelines for Genetic/Familial High-Risk Assessment: Breast, Ovarian, and Pancreatic V1.2020 (21) suggest insufficient evidence for risk-reducing mastectomy and management based on family history.

c Excluded p. I157T carriers.

**Table 5.** Sensitivity analysis: adjusted odds ratios and 95% confidence intervals for gene associations with contralateral breast cancer among Caucasian patients (n = 51 222)

Genes or gene group	PBC			CBC			AOR (95% CI)^a^	*P*
	n_carrier_	n_tested_	Mutation prevalence, %	n_carrier_	n_tested_	Mutation prevalence, %		
Group^b^								
All actionable genes	2753	34 648	7.95	280	2109	13.28	1.72 (1.50 to 1.96)	<.001
RRM^+^	1546	39 383	3.93	151	2275	6.64	1.71 (1.43 to 2.03)	<.001
RRM^-^	1518	34 648	4.38	143	2109	6.78	1.52 (1.26 to 1.81)	<.001
Genes								
ATM	489	37 742	1.30	36	2198	1.64	1.18 (0.82 to 1.63)	.36
BRCA1	677	47 884	1.41	73	2588	2.82	1.94 (1.50 to 2.47)	<.001
BRCA2	784	47 884	1.64	63	2588	2.43	1.55 (1.19 to 2.00)	.001
CHEK2^c^	741	37 293	1.99	81	2178	3.72	1.81 (1.42 to 2.28)	<.001
CDH1	—	—	—	—	—	—	—	—
NBN	75	35 723	0.21	12	2124	0.56	2.79 (1.43 to 4.97)	.001
NF1	—	—	—	—	—	—	—	—
PALB2	358	39 561	0.90	33	2279	1.45	1.53 (1.05 to 2.17)	.02
PTEN	—	—	—	—	—	—	—	—
TP53	99	47 852	0.21	14	2587	0.54	2.10 (1.14 to 3.58)	.01

a Odds ratios estimated from models adjusted for age at diagnosis of first breast cancer, histology of the first breast cancer, personal history of other cancer, presence of first- or second-degree relative with breast cancer, and prior *BRCA1* and *BRCA2* genetic testing. “—” indicates gene sets or specific genes for which there were <5 carriers in any group. AOR = adjusted odds ratio; CBC = contralateral breast cancer; CI = confidence interval; PBC = primary breast cancer; RRM = risk-reducing mastectomy.

b RRM^+^: the set of genes recognized by NCCN Guidelines for Genetic/Familial High-Risk Assessment: Breast, Ovarian, and Pancreatic V1.2020 (21) as appropriate for discussion of risk-reducing mastectomy; RRM^-^: the set of genes for which NCCN Guidelines for Genetic/Familial High-Risk Assessment: Breast, Ovarian, and Pancreatic V1.2020 (21) suggest insufficient evidence for risk-reducing mastectomy and management based on family history.

c Excluded p. I157T carriers.

In African Americans, similar trends were observed for P/LP variants, although 95% confidence intervals were wider because of reduced sample size (Table 4). In fully adjusted models, African Americans with P/LP variants were 1.88 (95% CI = 1.36 to 2.56) times as likely to have SBC as noncarriers. Odds ratios for RRM+ P/LP variants were 2.26 (95% CI = 1.65 to 3.07) and 1.09 (95% CI = 0.47 to 2.18) for RRM^-^ P/LP variants. The most statistically significant gene-specific associations were for *PALB2* (OR = 2.75, 95% CI = 1.43 to 5.00; *P* = .001) and *BRCA2* (OR = 2.01, 95% CI = 1.29 to 3.02; *P* = .001), followed by *TP53* and *BRCA1* (OR = 3.26 and 1.79, respectively). With extremely low carriers, *CHEK2* was not statistically significantly associated with SBC in African Americans. Similar trends were also observed for Hispanic women; those with P/LP variants were 1.66 (95% CI = 1.02 to 2.58) times as likely to have SBC as noncarriers. However, none of the gene-specific associations with SBC were statistically significant among Hispanic women, except *BRCA1*, with an odds ratio of 2.21 (95% CI = 1.25 to 3.70). In Asian and Ashkenazi Jewish women, no statistically significant associations were observed, although sample size was too low for stable inferences.

**Table 3.** Matched analysis: odds ratios and 95% confidence intervals for gene associations with SBC among Caucasian patients matching for years between age at PBC and age at genetic testing

Genes or gene group	PBC		SBC		OR (95% CI)^a^	*P*
	n_carrier_	n_tested_	n_carrier_	n_tested_		
Group^b^						
All actionable genes	1126	13 780	423	3615	1.42 (1.25 to 1.63)	<.001
RRM^+^	603	15 503	234	4095	1.47 (1.24 to 1.75)	<.001
RRM^-^	626	13 780	226	3615	1.33 (1.12 to 1.58)	.001
Genes						
ATM	217	15 065	65	3957	0.94 (0.69 to 1.27)	.69
BRCA1	251	19 754	115	4859	1.93 (1.51 to 2.48)	<.001
BRCA2	290	19 754	110	4859	1.42 (1.11 to 1.82)	.005
CHEK2^c^	317	14 814	128	3908	1.59 (1.27 to 2.01)	<.001
CDH1	—	—	—	—	—	—
NBN	37	14 344	13	3769	1.45 (0.71 to 2.96)	.31
NF1	—	—	—	—	—	—
PALB2	162	15 611	49	4109	1.26 (0.88 to 1.79)	.21
PTEN	—	—	—	—	—	—
TP53	33	19 732	15	4855	1.65 (0.81 to 3.37)	.17

a Odds ratios estimated using conditional logistic regression, matching on age at diagnosis of first breast cancer, histology of the first breast cancer, personal history of other cancer, presence of first- or second-degree relative with breast cancer, prior *BRCA1* and *BRCA2* genetic testing, time between age at first breast cancer and age at genetic testing. “—” indicates gene sets or specific genes for which there were <5 carriers in any group. CBC = contralateral breast cancer; CI = confidence interval; OR = odds ratio; PBC = primary breast cancer; RRM = risk-reducing mastectomy; SBC = second breast cancer.

b RRM^+^: the set of genes recognized by NCCN Guidelines for Genetic/Familial High-Risk Assessment: Breast, Ovarian, and Pancreatic V1.2020 (21) as appropriate for discussion of risk-reducing mastectomy; RRM^-^: the set of genes for which NCCN Guidelines for Genetic/Familial High-Risk Assessment: Breast, Ovarian, and Pancreatic V1.2020 (21) suggest insufficient evidence for risk-reducing mastectomy and management based on family history.

c Excluded p. I157T carriers.

### Sensitivity Analyses

Results were generally similar when women with a SBC occurring within 1 year of the PBC (potentially synchronous breast cancer diagnoses) were included in the SBC group. However, there was no statistically significant difference between the 2 groups in mean age at PBC diagnosis (49.5 [SD = 11.5] vs 49.4 [SD = 10.9] years; *P* = .56). Likewise, the observed genetic associations among Caucasian women that included synchronous SBC (Supplementary Table 6, available online) were also similar to those found in the restricted set of the primary analysis. In the fully adjusted model, women with P/LP variants were 1.50 times as likely (95% CI = 1.37 to 1.64) to have SBC than those without P/LP variants. Odds ratios for RRM^+^ and RRM^-^ P/LP variants were 1.47 and 1.41, respectively (Supplementary Table 6, available online).

When the SBC group was further restricted to only those individuals with known CBC, all previously observed associations were stronger in magnitude and highly statistically significant despite the decreased sample size (Table 5). In models adjusted for all potential confounders, Caucasian women carrying P/LP variants in clinically actionable genes were 1.72 (95% CI = 1.50 to 1.96) times as likely to have CBC as noncarriers. Similarly, Caucasian women with P/LP in RRM+ or RRM- categories were 1.71 (95% CI = 1.43 to 2.03) or 1.52 (95% CI = 1.26 to 1.81) times as likely to have CBC, respectively. As previously observed, *BRCA1*, *CHEK2*, and *BRCA2* were the genes with the most prevalent P/LP variants associated with known CBC (OR = 1.94, 1.81, and 1.55, respectively; all *P* < .001). Specifically, *CHEK2* c.1100delC carriers were 1.84 (95% CI = 1.34 to 2.47) times as likely to have CBC as noncarriers. Furthermore, *NBN*, *TP53*, and *PALB2* were also statistically significantly associated with CBC (OR = 2.79, 2.10, and 1.53, respectively; *P* range from .001 to .02).

**Table 4.** Adjusted odds ratios and 95% confidence intervals for gene associations with SBC by racial and ethnic group

Racial/ethnic group	PBC			SBC			AOR (95% CI)^a^	*P*
	n_carrier_	n_tested_	Mutation prevalence, %	n_carrier_	n_tested_	Mutation prevalence, %		
African American^b^								
All actionable genes	273	3876	7.04	56	440	12.73	1.88 (1.36 to 2.56)	<.001
RRM^+^	263	4620	5.69	60	505	11.88	2.26 (1.65 to 3.07)	<.001
RRM^-^	60	3876	1.55	8	440	1.82	1.09 (0.47 to 2.18)	.83
ATM	36	4312	0.83	5	481	1.04	1.11 (0.38 to 2.65)	.83
BRCA1	135	5727	2.36	29	642	4.52	1.79 (1.15 to 2.69)	.007
BRCA2	136	5727	2.37	28	642	4.36	2.01 (1.29 to 3.02)	.001
CHEK2	—	—	—	—	—	—	—	—
CDH1	—	—	—	—	—	—	—	—
NBN	—	—	—	—	—	—	—	—
NF1	—	—	—	—	—	—	—	—
PALB2	45	4634	0.97	14	507	2.76	2.75 (1.43 to 5.00)	.001
PTEN	—	—	—	—	—	—	—	—
TP53	11	5725	0.19	6	642	0.93	3.26 (1.08 to 9.00)	.03
Hispanic^b^								
All actionable genes	240	3081	7.79	24	190	12.63	1.66 (1.02 to 2.58)	.08
RRM^+^	225	3658	6.15	21	218	9.63	1.55 (0.93 to 2.46)	.59
RRM^-^	63	3082	2.04	5	190	2.63	1.29 (0.44 to 2.99)	.26
ATM	—	—	—	—	—	—	—	—
BRCA1	129	4592	2.81	17	254	6.69	2.21 (1.25 to 3.70)	.004
BRCA2	—	—	—	—	—	—	—	—
CHEK2	—	—	—	—	—	—	—	—
CDH1	—	—	—	—	—	—	—	—
NBN	—	—	—	—	—	—	—	—
NF1	—	—	—	—	—	—	—	—
PALB2	44	3675	1.20	5	218	2.29	1.98 (0.67 to 4.67)	.16
PTEN	—	—	—	—	—	—	—	—
TP53	—	—	—	—	—	—	—	—
Asian^b^								
All actionable genes	163	2624	6.21	13	234	5.56	0.97 (0.52 to 1.69)	.93
RRM^+^	152	3058	4.97	15	253	5.93	1.34 (0.74 to 2.26)	.30
RRM^-^	—	—	—	—	—	—	—	—
ATM	—	—	—	—	—	—	—	—
BRCA1	70	3682	1.90	9	288	3.13	1.80 (0.82 to 3.50)	.11
BRCA2	—	—	—	—	—	—	—	—
CHEK2	—	—	—	—	—	—	—	—
CDH1	—	—	—	—	—	—	—	—
NBN	—	—	—	—	—	—	—	—
NF1	—	—	—	—	—	—	—	—
PALB2	—	—	—	—	—	—	—	—
PTEN	—	—	—	—	—	—	—	—
TP53	—	—	—	—	—	—	—	—
Ashkenazi Jewish^b^								
All actionable genes	261	3132	8.33	31	383	8.09	0.87 (0.58 to 1.28)	.49
RRM^+^	107	3445	3.11	11	414	2.66	0.77 (0.38 to 1.41)	.43
RRM^-^	172	3132	5.49	22	383	5.74	0.94 (0.57 to 1.46)	.78
ATM	31	3336	1	6	405	1	1.71 (0.63 to 3.90)	.24
BRCA1	73	3996	2	9	468	2	0.85 (0.39 to 1.64)	.64
BRCA2	—	—	—	—	—	—	—	—
CHEK2^c^	129	3310	3.90	16	401	3.99	0.93 (0.53 to 1.55)	.80
CDH1	—	—	—	—	—	—	—	—
NBN	—	—	—	—	—	—	—	—
NF1	—	—	—	—	—	—	—	—
PALB2	—	—	—	—	—	—	—	—
PTEN	—	—	—	—	—	—	—	—
TP53	—	—	—	—	—	—	—	—

a Odds ratios estimated from models adjusted for age at diagnosis of first breast cancer, histology of the first breast cancer, personal history of other cancer, presence of first- or second-degree relative with breast cancer, and prior *BRCA1* and *BRCA2* genetic testing. “—” indicates gene sets or specific genes for which there were less than 5 carriers in any group. AOR = adjusted odds ratios; CI = confidence interval; PBC = primary breast cancer; RRM = risk-reducing mastectomy; SBC = second breast cancer.

b RRM^+^: the set of genes recognized by NCCN Guidelines for Genetic/Familial High-Risk Assessment: Breast, Ovarian, and Pancreatic V1.2020 (21) as appropriate for discussion of risk-reducing mastectomy; RRM^-^: the set of genes for which NCCN Guidelines for Genetic/Familial High-Risk Assessment: Breast, Ovarian, and Pancreatic V1.2020 (21) suggest insufficient evidence for risk-reducing mastectomy and management based on family history.

c Excluded p. I157T carriers.

When cases with more than 2 breast cancers were excluded from the SBC group, previously observed associations remain for SBC-only Caucasian women (Supplementary Table 7, available online). For clinically actionable genes, those carrying P/LP variants were 1.40 (95% CI = 1.25 to 1.56) times as likely to have SBC than noncarriers. SBC-only was also associated with P/LP variants in RRM+, RRM-, *BRCA1*, *CHEK2*, and *PALB2* (OR = 1.36, 1.33, 1.46, 1.52, and 1.38, respectively; all *P* ≤ .001).

Additionally, exclusion of stage 0 ductal carcinoma in situ cases from both the PBC and SBC groups had little impact on the association between SBC and P/LP carrier status (OR = 1.43, 95% CI = 1.27 to 1.60).

## Discussion

In this enrichment analysis of genetic test results and detailed clinical histories from a large multi-ethnic cohort of patients tested at a single laboratory, we found that across Caucasian, Hispanic, and African American women, those carrying a P/LP variant in a clinically actionable gene were 44%-87.0% more likely to have SBC after adjusting for multiple potential confounders such as family history, prior *BRCA1/2* testing, and age at PBC diagnosis. Within each racial and ethnic group, mutated genes deemed eligible for RRM recommendation had similar effect sizes to those considered to have insufficient evidence for RRM recommendation, indicating that these findings were not driven solely by mutations in *BRCA1/2*. *BRCA1* and *CHEK2* were most prevalent among Caucasian women with SBC, whereas *PALB2* and *BRCA2* were most prevalent among African American women. Importantly, our sensitivity analyses comparing a subset of SBC women with clinically reported CBC to women with PBC yielded stronger associations with these genes despite reduced sample size. It is important to note that this is a retrospective analysis and does not directly link these P/LP variants with SBC. Future prospective longitudinal studies are needed to confirm a causal relationship between these non-*BRCA1/2* P/LP variants and SBC and to determine the absolute risk of SBC, similar to studies on *BRCA1/2* carriers.

Our findings are largely consistent with the observations of smaller retrospective studies, such as the Women’s Environment, Cancer, and Radiation Exposure (WECARE) study. P/LP variants in *BRCA1* and *BRCA2* have been reported to be associated with CBC in several studies, including WECARE (10-14). WECARE observed no statistically significant main effect of *ATM* on CBC, although such variation may enhance the deleterious effects of radiation exposure (23,24). Likewise, we did not observe an association between *ATM* and SBC or between *ATM* and confirmed CBC. In our study, *CHEK2* was also strongly associated with SBC and CBC in contrast to the previous WECARE study (25). Interestingly, most CBC studies evaluating *CHEK2* have focused on the c.1100delC variant (15,25,26), the results of which have been inconsistent; 1 study observed associations with CBC incidence and long-term survival (26), whereas the WECARE consortium reported no association but acknowledged small sample size and limited power (25). We confirmed that carriers of the c.1100delC *CHEK2* variant were 1.5 times as likely to have SBC than noncarriers; the odds of SBC increased to approximately 1.8 in sensitivity analyses restricting to women with known CBC, in line with effect sizes reported previously (25).

Our findings also shed new light on other gene-based associations with CBC. For example, WECARE investigators previously reported a statistically significant association between *PALB2* and CBC in a sample of 1124 women (93% Caucasian), with a marginal *P* value (*P* = .04) based on only 5 carriers of *PALB2* truncating mutations among women with CBC vs no carriers among women with only 1 primary (16). Having no control carriers further precluded WECARE investigators from estimating *PALB2*-associated risk for CBC. Our results from a cohort of nearly 90 000 women suggest a modestly higher prevalence of *PALB2* among Caucasian women with CBC compared with PBC and substantially increased prevalence among African American women (27).

Our study has limitations. Despite our large sample size, we were precluded from reporting gene-specific associations in some racial and ethnic groups because of the extremely low frequency of P/LP variants in these genes. We also lacked sufficient power to make inferences on most variant-specific effects. Hence, our analyses and interpretation of findings are based on gene-level enrichment of rare pathogenic variants, which can be confounded by allelic heterogeneity (28). Women with hormone receptor–negative PBC are at higher risk of SBC (29-32), and tumors in *BRCA1* carriers are more likely to be hormone negative (33); an analysis of those patients with complete ER status of their PBC showed identical findings to our analysis without adjusting for ER status. Given the high number of missing values, we could not adjust for specific tumor subtypes such as triple-negative breast cancer or HER2-positive tumors. Hormonal therapy and chemotherapy have been shown to decrease risk of CBC (34); however, our study data did not include treatment information for PBC, so we were unable to assess treatment effects and their potential interactions with P/LP variants and SBC. However, we would not expect P/LP carriers to receive different systemic treatments than noncarriers that would have impacted SBC. Lastly, we also did not have information on whether patients underwent a bilateral mastectomy at the time of PBC diagnosis, which is an important confounder because bilateral mastectomy rates have been increasing over the past decade (35-38). However, studies have shown patient age at diagnosis is a larger driver of the decision to undergo bilateral mastectomy than genetic risk factors (39,40). When we examined associations in women younger than 50 years and conducted an age-matched analysis, our initial estimates did not change. Because bilateral mastectomy would reduce the risk of a subsequent breast cancer, our estimates of SBC risk for women with P/LP variants may be conservative.

Caution should be exercised in using these findings to dictate clinical management. Our findings showed an association between some non-*BRCA1/2* P/LP genes and SBC. These findings could be due to confounding, that is, “confounding by indication,” because of ascertainment differences between cases and controls (eg, NCCN Guidelines for SBC patients allow for older diagnosis age than a patient from our PBC group). Nevertheless, we adjusted for many confounders associated with increased risk of a SBC such as family history, personal history of other cancers, and age at diagnosis based on available information using the adjusted logistic regression models, matched analysis, and stratified analysis. However, future studies are needed to confirm our findings before clinical recommendations can be made.

In conclusion, our results show that in patients carrying a P/LP variant, the odds of having SBC are higher than in noncarriers. These findings underscore the need for future studies examining the role of non-*BRCA1/2* genes in SBC and CBC to help inform the complex decision-making process that physicians and patients must navigate when results of multigene panel testing are returned for a patient with a new breast cancer.

## Funding

This work was supported by the Harold and Jane Perlman Family Foundation (in memory of Joni Perlman Rosenberg).

## Notes

**Role of the funder:** The funder had no role in the design of the study; the collection, analysis, and interpretation of the data; the writing of the manuscript; and the decision to submit the manuscript for publication.

**Disclosures:** The authors declare no conflicts of interest. Clifford, Li, LaDuca, Gutierrez, and Black were employed at Ambry Genetics, a commercial lab, at the time of the study.

**Disclaimers:** We have no disclaimers to report.

**Acknowledgments:** We would like to acknowledge Brice Sarver for their help in data collection and constructing Table 1.

**Prior presentations:** This study was presented at the 2018 San Antonio Breast Cancer Symposium (poster presentation), San Antonio, Texas, December 2018.

**Author contributions:** All authors participated in design of the study and writing of the manuscript. JC, MB, and SL performed the analysis of the results.

## Data Availability

The data that support the findings of this study are available from the corresponding author (KAY) upon reasonable request.

## Supplementary Material

pkaa094_Supplementary_Data
