# Supplementary material for: Prevalence of Germline Pathogenic and Likely Pathogenic Variants in Patients With Second Breast Cancers
Source: JNCI Cancer Spectr. 2020 Oct 26;4(6):pkaa094. doi: 10.1093/jncics/pkaa094 (PMC7771422; doi:10.1093/jncics/pkaa094)
Supplement: pkaa094_Supplementary_Data [file pkaa094_supplementary_data.pdf]

SUPPLEMENTARY MATERIALS

Supplementary Table 1. Genes included in multi-gene panels

Multi-Gene Panel Test	Genes Included
BRCAplus	*BRCA1, BRCA2, CDH1, PALB2*[1]*, PTEN, STK11*[2]*, TP53*
BRCAplus Expanded	*ATM, BRCA1, BRCA2, CDH1, CHEK2, PALB2, PTEN, TP53*
BreastNext	*ATM, BARD1, BRCA1*[3]*, BRCA2*[3]*, BRIP1, CDH1, CHEK2, MRE11A, MUTYH, NBN, NF1*[4]*, PALB2, PTEN, RAD50, RAD51C, RAD51D*[4]*, STK11*[2]*, TP53*
GYNplus	*BRCA1, BRCA2, BRIP1*[7]*, EPCAM*[5]*, MLH1, MSH2, MSH6, PALB2*[7]*, PMS2, PTEN, RAD51C*[7]*, RAD51D*[7]*, TP53*
OvaNext	*ATM, BARD1, BRCA1*[3]*, BRCA2*[3]*, BRIP1, CDH1, CHEK2, EPCAM*[5]*, MLH1, MRE11A, MSH2, MSH6, MUTYH, NBN, NF1*[4]*, PALB2, PMS2, PTEN, RAD50, RAD51C, RAD51D*[4]*, SMARCA4*[6]*, STK11, TP53*
PancNext	*APC, ATM, BRCA1, BRCA2, CDKN2A, EPCAM*[5]*, MLH1, MSH2, MSH6, PALB2, PMS2, STK11, TP53*
CancerNext	*APC, ATM, BARD1, BRCA1*[3]*, BRCA2*[3]*, BRIP1, BMPR1A, CDH1, CDK4*[4]*, CDKN2A*[4]*, CHEK2, EPCAM*[5]*, GREM1*[5,6]*, MLH1, MRE11A, MSH2, MSH6, MUTYH, NBN, NF1*[4]*, PALB2, PMS2, POLD1*[6]*, POLE*[6]*, PTEN, RAD50, RAD51C, RAD51D*[4]*, SMAD4, SMARCA4*[6]*, STK11, TP53*
CancerNext-Expanded	*APC, ATM, BAP1*[6]*, BARD1, BRCA1, BRCA2, BRIP1, BMPR1A, CDH1, CDK4, CDKN2A, CHEK2, EPCAM*[5]*, FH, FLCN, GREM1*[5,6]*, MAX, MEN1, MET, MITF*[8]*, MLH1, MRE11A, MSH2, MSH6, MUTYH, NBN, NF1, PALB2, PMS2, POLD1*[6]*, POLE*[6]*, PTEN, RAD50, RAD51C, RAD51D, RET, SDHA, SDHAF2, SDHB, SDHC, SDHD, SMAD4, SMARCA4*[6]*, STK11, TMEM127, TP53, TSC1, TSC2, VHL*

[1]*PALB2* included for panels ordered on or after 10/1/2015

[2]*STK11* removed for panels orders authorized on or after 8/1/14

[3]*BRCA1* and *BRCA2* included for panels ordered on or after 6/13/13

[4]*NF1, RAD51D, CDKN2A,* and *CDK4* included for panels ordered on or after 10/18/13

[5]*EPCAM* and *GREM1* include reporting of selected gross deletions/duplications only

[6]*BAP1, GREM1, POLD1, POLE, SMARCA4, FH* and *MEN1* included for panels ordered on or after 5/18/15

[7]*BRIP1*, *PALB2*, *RAD51C*, and *RAD51D* included for panels ordered on or after 6/1/16

[8]For *MITF* only the status of the c.952G>A (p.E318K) alteration is analyzed and reported.

Supplementary Table 2. Patient Demographic Factors by Race/Ethnicity

	Caucasian n=53,122	African American n=6,369	Asian n=3,970	Ashkenazi Jewish n=4,464	Hispanic n=4,846	Other n=10,507	P-value
Mean±SD age at testing (years)	55.4 ± 12.1	50.8 ± 11.8	49.0 ± 11.4	59.1 ± 12.1	49.5 ± 11.4	52.8 ± 12.1	<.001
Mean±SD age at diagnosis of first breast cancer primary (years)	50.0 ± 11.3	46.9 ± 11.1	45.4 ± 10.2	52.5 ± 11.6	46.0 ± 10.6	48.4 ± 11.3	<.001
Mean±SD Years between breast primaries	12.6 ± 7.8	11.8 ± 7.6	11.4 ± 6.6	12.4 ± 7.6	10.8 ± 7.2	12.1 ± 7.5	
Panel Test (%)							<.001
BRCAplus BRCAplus Expanded	11656 (21.9)	1815 (28.5)	995 (25.1)	759 (17.0)	1381 (28.5)	2317 (22.1)	
GYNplus	1388 (2.6)	146 (2.3)	66 (1.7)	84 (1.9)	111 (2.3)	231 (2.2)	
BreastNext	16839 (31.7)	2148 (33.7)	1220 (30.7)	1368 (30.6)	1577 (32.5)	3465 (33.0)	
OvaNext	8795 (16.6)	921 (14.5)	882 (22.2)	823 (18.4)	812 (16.8)	1650 (15.7)	
PancNext	159 (0.3)	13 (0.2)	4 (0.1)	16 (0.4)	11 (0.2)	28 (0.3)	
CancerNext/ CancerNext Expanded	14277 (26.9)	1325 (20.8)	801 (20.2)	1413 (31.7)	954 (19.7)	2813 (26.8)	
Other	1 (0.0)	0 (0.0)	0 (0.0)	0 (0.0)	0 (0.0)	0 (0.0)	
Previously tested for *BRCA1/2* (%)							<.001
Yes	9077 (17.1)	726 (11.4)	597 (15.0)	1454 (32.6)	699 (14.4)	1695 (16.1)	
No	41935 (78.9)	5383 (84.5)	3173 (79.9)	2396 (53.7)	3952 (81.6)	8357 (79.5)	
Unknown	2110 (4.0)	260 (4.1)	200 (5.0)	614 (13.8)	195 (4.0)	455 (4.3)	
Patient-reported second primary type (%)							<.001
Contralateral/bilateral	2588 (4.9)	323 (5.1)	170 (4.3)	229 (5.1)	138 (2.8)	432 (4.1)	
Ipsilateral	547 (1.0)	61 (1.0)	25 (0.6)	52 (1.2)	22 (0.5)	76 (0.7)	
Not provided	2101 (4.0)	258 (4.1)	93 (2.3)	187 (4.2)	94 (1.9)	331 (3.2)	

Personal history of other cancer (%)							<.001
Yes	6712 (12.6)	475 (7.5)	269 (6.8)	650 (14.6)	327 (6.7)	1057 (10.1)	
No	46410 (87.4)	5894 (92.5)	3701 (93.2)	3814 (85.4)	4519 (93.3)	9450 (89.9)	
Personal history of other cancers, type (%)							
Ovarian	1100 (2.1)	69 (1.1)	54 (1.4)	74 (1.7)	51 (1.1)	156 (1.5)	<.001
Endometrial	1152 (2.2)	90 (1.4)	50 (1.3)	99 (2.2)	63 (1.3)	174 (1.7)	<.001
Colorectal	807 (1.5)	85 (1.3)	34 (0.9)	66 (1.5)	36 (0.7)	138 (1.3)	<.001
Melanoma	1223 (2.3)	4 (0.1)	3 (0.1)	119 (2.7)	10 (0.2)	125 (1.2)	<.001
Pancreatic	221 (0.4)	19 (0.3)	6 (0.2)	25 (0.6)	10 (0.2)	28 (0.3)	<.001
Other	2589 (4.9)	198 (3.1)	121 (3.0)	296 (6.6)	150 (3.1)	485 (4.6)	<.001
Family history of any cancer (%)							
≥1 first degree relative	37601 (70.8)	3645 (57.2)	1937 (48.8)	3280 (73.5)	2481 (51.2)	6669 (63.5)	<.001
≥1 second/third degree relatives only	11256 (21.2)	1785 (28.0)	1052 (26.5)	872 (19.5)	1365 (28.2)	2674 (25.4)	
none	4265 (8.0)	939 (14.7)	981 (24.7)	312 (7.0)	1000 (20.6)	1164 (11.1)	
Family history of breast cancer (%)							
≥1 first degree relative	20224 (38.1)	2042 (32.1)	985 (24.8)	1697 (38.0)	1273 (26.3)	3450 (32.8)	<.001
≥1 second/third degree relatives only	17618 (33.2)	2043 (32.1)	878 (22.1)	1434 (32.1)	1277 (26.4)	3493 (33.2)	
none	15280 (28.8)	2284 (35.9)	2107 (53.1)	1333 (29.9)	2296 (47.4)	3564 (33.9)	
Family history of ovarian cancer (%)							
≥1 first degree relative	2915 (5.5)	287 (4.5)	169 (4.3)	204 (4.6)	244 (5.0)	501 (4.8)	<.001
≥1 second/third degree relatives only	5471 (10.3)	578 (9.1)	274 (6.9)	373 (8.4)	410 (8.5)	1011 (9.6)	
none	44736 (84.2)	5504 (86.4)	3527 (88.8)	3887 (87.1)	4192 (86.5)	8995 (85.6)	
Met testing criteria for *BRCA1/2*							<.001

Yes	47716 (89.8)	5929 (93.1)	3507 (88.3)	4462 (100.0)	4337 (89.5)	9376 (89.2)	
No	5406 (10.2)	440 (6.9)	463 (11.7)	2 (0.0)	509 (10.5)	1131 (10.8)	
Met testing criteria for Li-Fraumeni syndrome (%)							<.001
Yes	5812 (10.9)	654 (10.3)	370 (9.3)	413 (9.3)	470 (9.7)	1181 (11.2)	
No	47310 (89.1)	5715 (89.7)	3600 (90.7)	4051 (90.7)	4376 (90.3)	9326 (88.8)	

Supplementary Table 3. Tumor Characteristics for PBC and SBC groups.

	PBC n=75,550	SBC n=7,728	
Histology, first breast cancer			<.001
Invasive ductal carcinoma (IDC)	41949 (55.5)	3655 (47.3)	
Invasive lobular carcinoma (ILC)	4233 (5.6)	266 (3.4)	
IDC and ILC	652 (0.9)	128 (1.7)	
Ductal carcinoma *in situ* (DCIS)	11109 (14.7)	1274 (16.5)	
Other rare subtype	1767 (2.3)	187 (2.4)	
Not provided	15840 (21.0)	2218 (28.7)	
Histology, second breast cancer			
Invasive ductal carcinoma (IDC)	--	3496 (45.2)	
Invasive lobular carcinoma (ILC)	--	392 (5.1)	
IDC and ILC	--	127 (1.6)	
Ductal carcinoma *in situ* (DCIS)	--	1113 (14.4)	
Other rare subtype	--	178 (2.3)	
Not provided	--	2422 (31.3)	
Hormone receptor status, first breast cancer primary			
ER			-
Positive	40279 (53.3)	2788 (36.1)	
Negative	13547 (17.9)	1156 (15.0)	
Not provided	21724 (28.8)	3784 (49.0)	
PR			-
Positive	33823 (44.8)	2150 (27.8)	
Negative	16668 (22.1)	1293 (16.7)	
Not provided	25059 (33.2)	4285 (55.4)	
HER2			-
Positive	8078 (10.7)	410 (5.3)	
Negative	32552 (43.1)	2180 (28.2)	
Not provided	34920 (46.2)	5138 (66.5)	
TNBC			-
Yes	9052 (12.0)	721 (9.3)	
No	43410 (57.5)	3016 (39.0)	
Not provided	23088 (30.6)	3991 (51.6)	
Hormone receptor status, second breast cancer primary			
ER			-
Positive	--	3177 (41.1)	
Negative	--	1176 (15.2)	

	Not provided	--	3375 (43.7)	
PR				-
	Positive	--	2461 (31.8)	
	Negative	--	1569 (20.3)	
	Not provided	--	3698 (47.9)	
HER2				-
	Positive	--	488 (6.3)	
	Negative	--	2710 (35.1)	
	Not provided	--	4530 (58.6)	
TNBC				-
	Yes	--	809 (10.5)	
	No	--	3406 (44.1)	
	Not provided	--	3513 (45.5)	

Abbreviations: ER, estrogen receptor; PR: progesterone receptor; HER2: human epidermal growth factor; TNBC; triple-negative breast cancer.

Supplementary Table 4. Matched analysis: odds ratios (OR)[a] and 95% confidence intervals (CI) for gene associations with SBC among Caucasian patients

	PBC		SBC			
	$n_{Carrier}$	n_{Tested}	$n_{Carrier}$	n_{Tested}	OR[a] (95% CI)	p-value
Group[b]						
All actionable genes	2683	33721	464	3968	1.45 (1.30,1.61)	<.001
RRM$^+$	1526	38486	254	4457	1.41 (1.23,1.62)	<.001
RRM$^-$	1475	33721	248	3968	1.37 (1.19,1.58)	<.001
Genes						
ATM	477	36804	70	4310	1.14 (0.88,1.47)	.34
BRCA1	669	46913	124	5232	1.60 (1.31,1.95)	<.001
BRCA2	777	46913	112	5232	1.33 (1.09,1.63)	0.006
CHEK2[d]	725	36372	141	4258	1.61 (1.33,1.94)	<.001
CDH1[c]	--	--	--	--	--	--
NBN	73	34798	15	4126	1.73 (0.98,3.05)	.06
NF1[c]	--	--	--	--	--	--
PALB2	352	38656	55	4470	1.29 (0.96,1.73)	.09
PTEN[c]	--	--	--	--	--	--
TP53	93	46881	20	5228	1.52 (0.92,2.50)	.10

[a]Odds ratios estimated using conditional logistic regression, matching on age at diagnosis of first breast cancer, histology of the first breast cancer, personal history of other cancer, presence of first- or second-degree relative with breast cancer, prior *BRCA1* and *BRCA2* genetic testing.

[b] RRM$^+$: the set of genes recognized by NCCN Guidelines for Genetic/Familial High-Risk Assessment: Breast, Ovarian, and Pancreatic V1.2020[21] as appropriate for discussion of risk-reducing mastectomy; RRM$^-$: the set of genes for which NCCN Guidelines for Genetic/Familial High-Risk Assessment: Breast, Ovarian, and Pancreatic V1.2020[21] suggest insufficient evidence for risk-reducing mastectomy and management based on family history

[c] "—" indicates gene sets or specific genes for which there were <5 carriers in any group.

[d]excluded p.I157T carriers

Supplementary Table 5. Stratification by PBC diagnosis age (<50 and ≥50 yrs): Adjusted Odds Ratios (AOR)a and 95% Confidence Intervals (CI) for Gene Associations with SBC Among Caucasian Patients.

	PBC			SBC				
	$n_{Carrier}$	n_{Tested}	Mutation Prevalence	$n_{Carrier}$	n_{Tested}	Mutation Prevalence	AOR (95% CI)	p-value
			Diagnosis age at first BC <50 yr					
Group[b]								
All actionable genes	1600	17939	8.92%	320	2375	13.47%	1.56(1.37,1.78)	<.001
RRM[+]	930	20452	4.55%	188	2675	7.03%	1.62(1.37,1.90)	<.001
RRM[-]	862	17939	4.81%	161	2375	6.78%	1.38(1.15,1.64)	.0004
Genes								
ATM	287	19569	1.47%	43	2576	1.67%	1.04(0.74,1.43)	.81
BRCA1	462	25286	1.83%	108	3134	3.45%	1.95(1.57,2.42)	<.001
BRCA2	458	25286	1.81%	72	3134	2.30%	1.35 (1.04,1.73)	.02
CHEK2[d]	423	19361	2.18%	98	2552	3.84%	1.73(1.37,2.16)	<.001
CDH1[c]	--	--	--	--	--	--	--	--
NBN	39	18513	0.21%	8	2470	0.32%	1.55(0.67,3.19)	.26
NF1[c]	--	--	--	--	--	--	--	--
PALB2	188	20508	0.92%	37	2681	1.38%	1.47(1.01,2.07)	.04
PTEN[c]	--	--	--	--	--	--	--	--
TP53	78	25278	0.31%	13	3131	0.42%	1.08(0.57,1.90)	.79
			Diagnosis age at first BC ≥50 yr					
Group[b]								
All actionable genes	1153	16709	6.90%	145	1604	9.04%	1.32(1.10,1.58)	.003
RRM[+]	616	18931	3.25%	66	1793	3.68%	1.14(0.87,1.46)	.33
RRM[-]	656	16709	3.93%	88	1604	5.49%	1.39(1.09,1.74)	.006
Genes								
ATM	202	18173	1.11%	28	1744	1.61%	1.39(0.91,2.04)	.11
BRCA1	215	22598	0.95%	16	2104	0.76%	0.78 (0.45,1.25)	.33
BRCA2	326	22598	1.44%	40	2104	1.90%	1.40(0.99,1.92)	.05
CHEK2[d]	318	17932	1.77%	43	1716	2.51%	1.40(1.00, 1.91)	.04
CDH1[c]	--	--	--	--	--	--	--	--
NBN	36	17210	0.21%	7	1666	0.42%	2.03(0.82,4.31)	.09
NF1[c]	--	--	--	--	--	--	--	--
PALB2	170	19053	0.89%	18	1800	1.00%	1.09(0.64,1.73)	.73
PTEN[c]	--	--	--	--	--	--	--	--

TP53	21	22574	0.09%	7	2103	0.33%	3.25 (1.27,7.39)	.008

[a]Odds ratios estimated from models adjusted for age at diagnosis of first breast cancer, histology of the first breast cancer, personal history of other cancer, presence of first- or second-degree relative with breast cancer, and prior *BRCA1* and *BRCA2* genetic testing.

[b] RRM$^+$: the set of genes recognized by NCCN Guidelines for Genetic/Familial High-Risk Assessment: Breast, Ovarian, and Pancreatic V1.2020[21] as appropriate for discussion of risk-reducing mastectomy; RRM$^-$: the set of genes for which NCCN Guidelines for Genetic/Familial High-Risk Assessment: Breast, Ovarian, and Pancreatic V1.2020[21] suggest insufficient evidence for risk-reducing mastectomy and management based on family history

[c] "—" indicates gene sets or specific genes for which there were <5 carriers in any group.

[d]excluded p.I157T carriers

Supplementary Table 6. Sensitivity Analysis for Cases with a Second Breast Cancer Diagnosed within One Year of Diagnosis: Adjusted odds ratios (AOR)[a] and 95% confidence intervals (CI) for gene associations with SBC among Caucasian patients (n=55706)

	PBC			SBC				
	$n_{Carrier}$	n_{Tested}	Mutation Prevalence	$n_{Carrier}$	n_{Tested}	Mutation Prevalence	AOR (95% CI)	p-value
Group[b]								
All actionable genes	2753	34648	7.95%	679	5889	11.53%	1.50 (1.37,1.64)	<.001
RRM+	1546	39383	3.93%	364	6662	5.46%	1.44 (1.28,1.62)	<.001
RRM-	1518	34648	4.38%	367	5890	6.23%	1.41 (1.25,1.59)	<.001
Genes								
ATM	489	37742	1.30%	100	6422	1.56%	1.16 (0.92,1.43)	.20
BRCA1	677	47884	1.41%	164	7822	2.10%	1.54 (1.29,1.82)	<.001
BRCA2	784	47884	1.64%	158	7822	2.02%	1.31 (1.10,1.55)	.002
CHEK2[c]	741	37293	1.99%	208	6346	3.28%	1.62 (1.39,1.90)	<.001
CDH1	29	46409	0.06%	6	7632	0.08%	1.28 (0.48,2.91)	.58
NBN	75	35723	0.21%	24	6145	0.39%	1.90 (1.17,2.97)	.007
NF1	42	34656	012%	7	5891	0.12%	0.99 (0.40,2.07)	.97
PALB2	358	39561	0.90%	76	6684	1.14%	1.23 (0.95,1.57)	.11
PTEN	31	47704	0.06%	14	7803	0.18%	2.71 (1.39,5.01)	.002
TP53	99	47852	0.21%	26	7816	0.33%	1.45 (0.92,2.20)	.10

[a]Odds ratios estimated from models adjusted for age at diagnosis of first breast cancer, histology of the first breast cancer, personal history of other cancer, presence of first- or second-degree relative with breast cancer, and prior *BRCA1* and *BRCA2* genetic testing.

[b] RRM^+: the set of genes recognized by NCCN Guidelines for Genetic/Familial High-Risk Assessment: Breast, Ovarian, and Pancreatic V1.2020[21] as appropriate for discussion of risk-reducing mastectomy; RRM^-: the set of genes for which NCCN Guidelines for Genetic/Familial High-Risk Assessment: Breast, Ovarian, and Pancreatic V1.2020[21] suggest insufficient evidence for risk-reducing mastectomy and management based on family history

[c]excluded p.I157T carriers

Supplementary Table 7. Sensitivity Analysis for cases with Second Breast Cancer only (excluded >2 BC): Adjusted odds ratios (AOR)[a] and 95% confidence intervals (CI) for gene associations with SBC among Caucasian patients

	PBC			SBC-only				
	$n_{Carrier}$	n_{Tested}	Mutation Prevalence	$n_{Carrier}$	n_{Tested}	Mutation Prevalence	AOR (95% CI)	p-value
Group[b]								
All actionable genes	2753	34648	7.95%	416	3676	11.32%	1.40 (1.25,1.56)	<.001
RRM^{+}	1546	39383	3.93%	226	4136	5.46%	1.36 (1.17,1.56)	<.001
RRM^{-}	1518	34648	4.38%	225	3676	6.12%	1.33 (1.15,1.54)	<.001
Genes								
ATM	489	37742	1.30%	68	3999	1.70%	1.19 (0.91,1.53)	.18
BRCA1	677	47884	1.41%	107	4853	2.20%	1.46 (1.18,1.79)	.0004
BRCA2	784	47884	1.64%	95	4853	1.96%	1.22 (0.97,1.50)	.075
CHEK2[c]	741	37293	1.99%	126	3951	3.19%	1.52 (1.24,1.83)	<.001
CDH1	--	--	--	--	--	--	--	--
NBN	75	35723	0.21%	13	3826	0.34%	1.64 (0.86,2.87)	.10
NF1	--	--	--	--	--	--	--	--
PALB2	358	39561	0.90%	53	4147	1.28%	1.38 (1.02,1.83)	.03
PTEN	--	--	--	--	--	--	--	--
TP53	99	47852	0.21%	19	4850	0.39%	1.38 (0.81,2.22)	.21

[a]Odds ratios estimated from models adjusted for age at diagnosis of first breast cancer, histology of the first breast cancer, personal history of other cancer, presence of first- or second-degree relative with breast cancer, and prior *BRCA1* and *BRCA2* genetic testing.

[b] RRM^{+}: the set of genes recognized by NCCN Guidelines for Genetic/Familial High-Risk Assessment: Breast, Ovarian, and Pancreatic V1.2020[21] as appropriate for discussion of risk-reducing mastectomy; RRM^{-}: the set of genes for which NCCN Guidelines for Genetic/Familial High-Risk Assessment: Breast, Ovarian, and Pancreatic V1.2020[21] suggest insufficient evidence for risk-reducing mastectomy and management based on family history

[c]excluded p.I157T carriers
